# Influencing factor analysis of family doctor contract service among older adults: evidence from China

**DOI:** 10.3389/fpubh.2024.1487365

**Published:** 2024-11-19

**Authors:** Shiyu Xie, Zihan Ni, Xiya Yang, Ningze Xu, Chengfang Zhu, Liting Huo, Xiuyuan Zhu, Xiaoguang Yang

**Affiliations:** ^1^School of Elderly Care Services and Management, Nanjing University of Chinese Medicine, Nanjing, China; ^2^School of Public Health, Fudan University, Shanghai, China; ^3^Wangying Community Health Service Center, Huai'an, China; ^4^Shanghai Publishing and Printing College, Shanghai, China; ^5^Chinese Hospital Development Institute, Shanghai Jiaotong University School of Medicine, Shanghai, China

**Keywords:** family doctor, family doctor contract service, older adults, primary healthcare, influencing factors

## Abstract

**Background:**

Family doctor contract services are essential to primary healthcare and play a significant role in improving the health of older adults. However, contract rates among older adults vary widely. Investigating the factors influencing contract rates is crucial for optimizing policies and increasing participation in family doctor services.

**Methods:**

This study used data from Wangying Community Health Service Center, encompassing 5,684 older adults in 2018. To address the endogeneity issue arising from sample selection bias, the study utilized a 1:1 nearest-neighbor matching method for counterfactual testing, balancing potential confounding factors between the contract and non-contract groups. Subsequently, multiple logistic regression analysis was performed on the matched data to explore the impact of gender, age, number of medical visits, and the number of chronic conditions on family doctor contract behavior.

**Results:**

Gender did not significantly impact contracting behavior. However, age, the number of medical visits, and the number of chronic diseases significantly influenced family doctor contract (*β* = 0.457, *p* < 0.01; *β* = 0.286, *p* < 0.05; *β* = −0.229, *p* < 0.1). An inverted U-shaped relationship was found between age and contracting behavior (*β* = −0.003, *p* < 0.01).

**Conclusion:**

The factors influencing older residents’ decisions to sign up for family doctor services are complex and diverse. The conclusion of the study provides valuable reference and guidance for policymakers to further improve the family doctor contracting system and optimize contracting strategies by considering the characteristics of different older adults.

## 1 Introduction

The prevalence of chronic non-communicable diseases is increasing, and healthcare needs of residents are expanding as more nations enter an aging society (1). Primary healthcare services, as the foundation of the healthcare system, can offer patients tailored and continuous medical services (2). Globally, high-quality primary healthcare systems have been demonstrated to significantly reduce disease incidence and mortality, increase healthcare service efficacy, and reduce health inequities, hence promoting the development of a healthy society (3–5). Different countries have evolved various primary healthcare models depending on their own circumstances. For instance, in the United Kingdom, the National Health Service (NHS) emphasizes the gatekeeping duty of general practitioners (6), while in the United States, community health centers focus on delivering basic medical care to low-income groups (6). Brazil generally offers primary healthcare services through family health teams and community health workers (7), while Japan’s healthcare system features universal health insurance and free medical treatment (8). Each primary healthcare paradigm has advantages and limitations, which can be broadly categorized into mandatory and non-mandatory primary care gatekeeping. Balancing the impacts of diverse models while boosting the appeal and utilization of basic healthcare services still necessitates the development of more specific policy optimization strategies.

In China, the primary healthcare system offers both general clinical treatment and basic public health services (9). The family doctor contract service is a core component of China’s primary healthcare system. Its primary purpose is to foster stable relationships between community residents and family doctors in order to deliver proactive, consistent, comprehensive, and affordable health accountability management models (10). The family doctor contract service was first launched in China in 2009 as an innovative and fundamental policy of the new healthcare reform and was fully implemented countrywide in 2016. It operates on the idea of voluntary contracts, prioritizing specific populations such as older adults and the disabled, offering basic medical care, public health, and agreed health management services (11). Family doctors, as crucial members of the primary healthcare system, perform a “dual gatekeeper” role in people’s health and healthcare expenditures. Their ultimate goal is to boost primary healthcare development, improve the tiered diagnosis and treatment system, as well as enhance the collaboration mechanism among medical institutions at all levels (12, 13). In contrast to the mandatory family doctor contracting and payment systems implemented in somecountries, residents in China sign 1-year contract with family doctors on their own free will. When the contract expires, residents can renew it with the same family doctor, select another doctor, or terminate it. To some extent, this system can objectively reflect the medical quality as well as diagnostic and treatment levels of family doctors (10). In contrast, the UK’s National Health Service (NHS) provides free healthcare through the “gatekeeper” role of general practitioners, where residents must obtain a referral from a general practitioner to access specialist care. While this system effectively controls healthcare costs and resource utilization, it lacks flexibility in patient choice. Japan, on the other hand, implements a universal health insurance system, where all citizens are required to participate, and medical expenses are shared by the government, employers, and individuals. Residents have the freedom to choose their medical institutions, but this freedom has led to fragmented and overutilized medical resources. Comparatively, China’s family doctor services offer greater convenience and rationality, yet the service utilization and quality under the current voluntary contract mechanism still have room for improvement (14, 15). Thus, further increasing the family doctor services contract and renewal rates among older residents can provide critical scientific evidence for improving family doctor service policies. However, under the voluntary contracting mechanism, the factors that influence contracting are complicated and diverse. Studies have shown that age influences older residents’ contracting behavior. Older adults typically face higher rates of chronic illness, functional decline, and disability, all of which increase their reliance on medical services (16). As individuals age, their healthcare needs become more complex, and they are more likely to require continuous, personalized, and accessible care—making the family doctor contract service particularly beneficial for them. Age is also an important factor influencing residents’ utilization of family doctor contract services (17). Older adults in different age groups may use the service differently. Younger older adults tend to use the service for continuous and stable management of their chronic diseases and for the prevention of other illnesses (18), while older adult individuals, due to more severe health conditions, require more targeted, convenient, and continuous medical care (19). For gender and family factors, researches indicate that men and high-income families are more likely to utilize family doctor contract services compared to women and low-income families. This disparity may be due to residents with higher social status having greater awareness of social information and government policies (20). Education level is also highly correlated with family doctor services contract rates, as residents with lower education levels find it challenging to understand medical information and related health suggestions (21). Older residents with better healthcare and self-rated health status use family doctor contract service more frequently (22), most likely because they have stronger economic capabilities, more access to medical services, as well as greater concern for their health. However, studies have found that the number of chronic diseases affects family doctor services contract rates among older individuals, which results in a higher willingness to contract among those with multiple chronic diseases (23). This is primarily because having multiple chronic diseases raises the risk of disability among older people, reduces their quality of life, and causes more adverse health outcomes than a single disease, severely impacting their physical and mental health (24). Additionally, choosing primary healthcare institutions for initial diagnosis, the number of visits, understanding of family doctor contract service as well as level of satisfaction and trust in family doctors all further influence the contract and renewal rates among older residents. Older individuals who prefer primary healthcare institutions for treatment may have a better understanding of the tiered diagnosis and treatment system, as well as the family doctor contract service policies (23). They may be more trusting and satisfied with primary healthcare doctors, making them more willing to contract with family doctors (25). Although primary healthcare doctors may not treat all health issues, the current smooth two-way referral channels allow family doctors to refer patients to higher-level medical institutions (23). Hence, as the number of visits increases, understanding and trust among older patients in family doctor services also raise, which helps improve contract and renewal rates.

More research is needed Under China’s voluntary family doctor service contract mechanism to improve the primary healthcare system reform. This study examines the family doctor contracting status of older individuals aged 60 and above in a specific city in China, analyzing key factors influencing contracting to provide scientific evidence for policymakers. It aims to promote the further development of family doctor contract services and reveal the main factors influencing patient healthcare behavior under the voluntary contracting and guided primary care models, providing reference for optimizing the primary healthcare system, improving health disparities, and reducing unreasonable medical expenses. The innovation of this study lies in utilizing firsthand medical visit data from the Wangying Community Health Service Center in Huai’an, where the implementation of the family doctor system is well-established and highly representative. This differentiates this study from those that use publicly available databases (26), and it will also make the research findings more authentic, targeted, and accurate, with policy recommendations that are more aligned with the actual needs of the residents. The focus of the research is on the older adults, a demographic specifically targeted by the policy and with significant demand for family doctor services, which distinguishes it from previous studies (27). This study aims to explore the impact of individual and health factors on their likelihood of contracting family doctors, and based on key influencing factors, propose policy improvement recommendations. Additionally, unlike those studies that directly use regression to explore the influencing factors of family doctor contract services (28), the study employs a combination of Propensity Score Matching (PSM) and multiple logistic regression to eliminate sample selection bias, thereby enhancing the rigor and depth compared to previous research.

## 2 Materials and methods

### 2.1 Participants

Huai’an, located in the central part of Jiangsu Province, is a typical densely populated city with rapid economic development. By the end of 2023, Huai’an had a population of older adults aged 60 and above totaling 1.2608 million, which constituted 23% of the city’s total registered population. Those aged 80 and above reached 178,000, accounting for 14.12% of the older population, a percentage that is higher than the national average (29). Huai’an was also among the first batch of Chinese cities to implement the family doctor contract service policy, adopting a “1 + X” model. “1” refers to the basic public health services provided free by the government, while “X” includes personalized fee-based services that residents can choose to add on a voluntary basis. The family doctor policy in Huai’an has been notably successful, with general population contract rates exceeding 50%, renewal rates over 80%, and key demographic contract rates around 80%, significantly surpassing the national average. Therefore, exploring the factors affecting the contracting rates of family doctor services for older adults in Huai’an will provide typical data for analysis. Micro-level individual data concerning family doctor contract services were sourced from the health information system of Wangying Community Health Service Center in Huai’an. Wangying Community Health Service Center was a pioneer in the family doctor contract system in Jiangsu Province as early as 2012, setting a model for family doctor services that has imposed significant influence both provincially and nationally. The health information system recorded detailed individual medical data for the entirety of 2018, offering micro-level insights for studying the factors influencing family doctor contract rates.

To precisely analyze the utilization of family doctor contract services by older adults aged 60 and above, the list of contracted residents was retrieved from the community health service center’s information system and matched with 2018 medical records to filter eligible older adults. The Wangying Community Health Service Center’s health information system exported a total of 49,434 records for 2018. After data cleaning and consolidation, incomplete medical records with missing key information were excluded. Ultimately, 5,684 valid samples of residents aged 60 and above were included in the study.

### 2.2 Measures

The study selected the contracting status of family doctor services as the dependent variable, used to measure whether older adults have established a formal service relationship with a family doctor. Specifically, if an older adult has signed a service agreement with a family doctor, this status is coded as 1; conversely, if an older adult has not yet signed up for family doctor services, this status is coded as 0.

In this study, gender, age, the number of medical visits, and the number of chronic diseases were selected as independent variables. Based on existing literature, to more meticulously assess the demand for and utilization patterns of family doctor services among different age groups of older adults, we categorized the sample data into six age groups with a five-year interval: 60–64 years, 65–69 years, 70–74 years, 75–79 years, 80–84 years, and 85 years and above. This grouping method helped identify potential differences in health needs, service preferences, and contracting behaviors among older adults at different stages of life. The number of medical visits and chronic diseases were directly extracted from the information system.

### 2.3 Statistical analysis

This study primarily investigated the effects of gender, age, the number of medical visits, and the number of chronic diseases on the contracting of family doctor services by older adults. Statistical analysis was conducted using Stata 16.0. Initially, descriptive statistics were performed on the variables collected through the questionnaire. Subsequently, the behavior of older individuals in enrolling in family doctor services is not random; it is influenced by various factors, including individual characteristics, which can lead to sample selection bias. To address this potential bias, the study employed propensity score matching (PSM) which involved generating random variables that follow a uniform distribution and propensity scores derived from a logistic regression model, to match the treatment group with the control group. After matching, only successfully matched observations were retained to minimize the selection bias caused by the non-randomness of the observational data, providing a more reliable basis for subsequent analyses. The study further employed multiple logistic regression to analyze the impact of various variables on the contracting of family doctor services by older adults and tested the significant associations between these factors and the contracting rate of family doctor services. The significance level for the tests was set at *α* = 0.05.

## 3 Results

### 3.1 Demographic characteristics

This study included a total of 5,684 older individuals aged 60 and above, with 2,398 males and 3,286 females, accounting for 50.17 and 49.88%, respectively. Regarding the contract status with family doctors, 50.00% older adults were contracted, while 50.00% were not. In terms of age distribution, there were 51.28 individuals aged 60–69 years, 35.10% individuals aged 70–79 years, 13.62% individuals aged 80 years and above. For the number of medical visits, the majority of older participants reported one to two visits, representing 53.78% of the total. Among the remaining respondents, 17.54% had three visits, while 28.68% had four or more visits. Regarding the number of chronic diseases, 30.67% of the older participants had a single chronic disease, while 69.33% of them suffered from multiple chronic comorbidities. Observationally, the contracting rate for family doctor services among older adults aged 65–69 was higher than for those aged 60–64 and 70–74. Specific data were presented in Table 1.

**Table 1:** Descriptive statistics of variables (*N* = 5,684).

Variable	Category	Total	Contracted	Uncontracted	Contracted rate (%)
Gender	Male	2,398	1,203	1,195	42.33
	Female	3,286	1,639	1,647	57.67
Age	60–64	1,381	659	722	23.19
	65–69	1,534	776	758	27.30
	70–74	1,133	619	514	21.78
	75–79	862	464	398	16.33
	80–84	481	227	254	7.99
	≥85	293	97	196	3.41
The number of medical visits	1	1,709	834	875	29.35
	2	1,348	644	704	22.66
	3	997	504	493	17.73
	4	697	338	359	11.89
	5	446	236	210	8.30
	≥6	487	286	201	10.06
The number of chronic diseases	1	1,743	853	890	30.01
	2	1,374	663	711	23.33
	3	1,006	511	495	17.98
	4	692	320	372	11.26
	5	440	243	197	8.55
	≥6	429	252	177	8.87

### 3.2 Propensity score matching

To minimize potential confounding factors, this study employed a logit model to calculate propensity scores, selecting four covariates: age, gender, the number of medical visits, and the number of chronic diseases. Using 1:1 nearest neighbor matching, samples with and without contracts were paired. Analysis using propensity score matching accounted for baseline differences between the treatment and control groups, enabling a more precise assessment of the relationships between contracting status and variables such as age, gender, and number of illnesses. Logistic regression analysis post-matching showed that age, gender, and number of illnesses significantly affected the likelihood of contracting. Specifically, an increase in the number of diseases was significantly positively correlated with the probability of contracting. Additionally, the effect of matching on the number of medical visits indicated that, compared to the control group, the average change in the number of visits for the treatment group was 0.1502 after matching, further revealing the potential impact of contracting status on medical behavior. Overall balance tests post-matching demonstrated good equilibrium between the treatment and control groups, validating the effectiveness of the study design and analytical methods.

### 3.3 Multiple logistic regression

**Figure 1:**
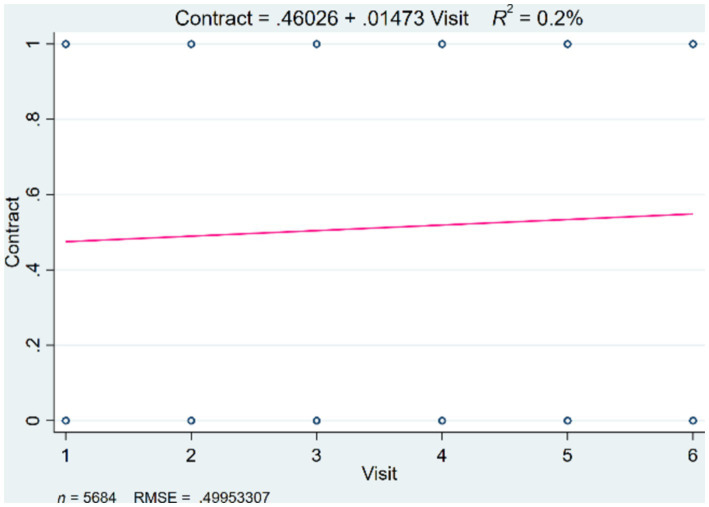
The relationship between the number of medical visits and contracting behavior.

This study utilized data after propensity score matching to perform multiple logistic regression on gender, age, the number of medical visits, and the number of chronic diseases to analyze their impacts on the contracting of family doctor services. Model 1 incorporated all the independent variables, while Model 2 was an enhanced version that includes an age-squared term (column 2). In the basic model, gender did not significantly influence the contracting of family doctor services, but age, the number of medical visits, and the number of chronic diseases had significant impacts. The results showed that younger age groups (*β* = −0.006, *p* < 0.1) had a positive correlation with the contracting rate, indicating that younger older individuals are more likely to sign up for family doctor services. The number of medical visits among older adults was also positively correlated with the contracting rate for family doctor services (*β* = 0.271, *p* < 0.05), indicating that the higher the likelihood and frequency of medical visits, the stronger the willingness to sign up for family doctor services. This study illustrated the relationship between the number of medical visits and contracting rate, as shown in Figure 1. Interestingly, the research found that an increase in the number of chronic diseases was negatively correlated with the contracting rate among older adults (*β* = −0.221, *p* < 0.1), meaning that a greater number of chronic diseases actually had a negative impact on the behavior of older adults in signing up for family doctor services. The study further explored the relationship between age and contracting behavior, finding that the positive coefficient for age groups indicated that, within a certain range, the probability of contracting increased with age. The introduction of the age-squared term in the enhanced model revealed a non-linear relationship between age and the contracting rate for family doctor services (*β* = −0.003, *p* < 0.01), indicating that the probability of contracting reached a maximum at a certain age point before beginning to decline. The coefficients for other variables remained consistent across the two models, as shown in Table 2.

**Table 2:** Results of multiple logistic regression.

	(1)	(2)
	Contracted	Contracted
Gender	−0.009 (0.054)	−0.004 (0.054)
Age	−0.006* (0.004)	0.457*** (0.062)
The number of medical visits	0.271** (0.122)	0.286** (0.123)
The number of chronic diseases	−0.221* (0.125)	−0.229* (0.126)
Square of age		−0.003*** (0.000)
_cons	0.298 (0.266)	−16.029*** (2.199)
*N*	5,684	5,684

Numbers in parentheses show standard errors. *p < 0.1, **p < 0.05, ***p < 0.01.

### 3.4 The impact of age on family doctor contracting services

Multiple logistic regression analysis revealed a significant nonlinear relationship between age and the family doctor contracting rates among older adults. To further investigate the relationship, we conducted a multiple logistic regression analysis, categorizing and squaring age. The results indicated that age groups have a significantly positive effect on the contracting behavior of family doctor services, with a coefficient of 0.107, while the squared term of age groups showed a significantly negative effect, with a coefficient of −0.001. The finding suggested that while the probability of contracting increases with age, it may be inhibited after reaching a certain age threshold.

**Figure 2:**
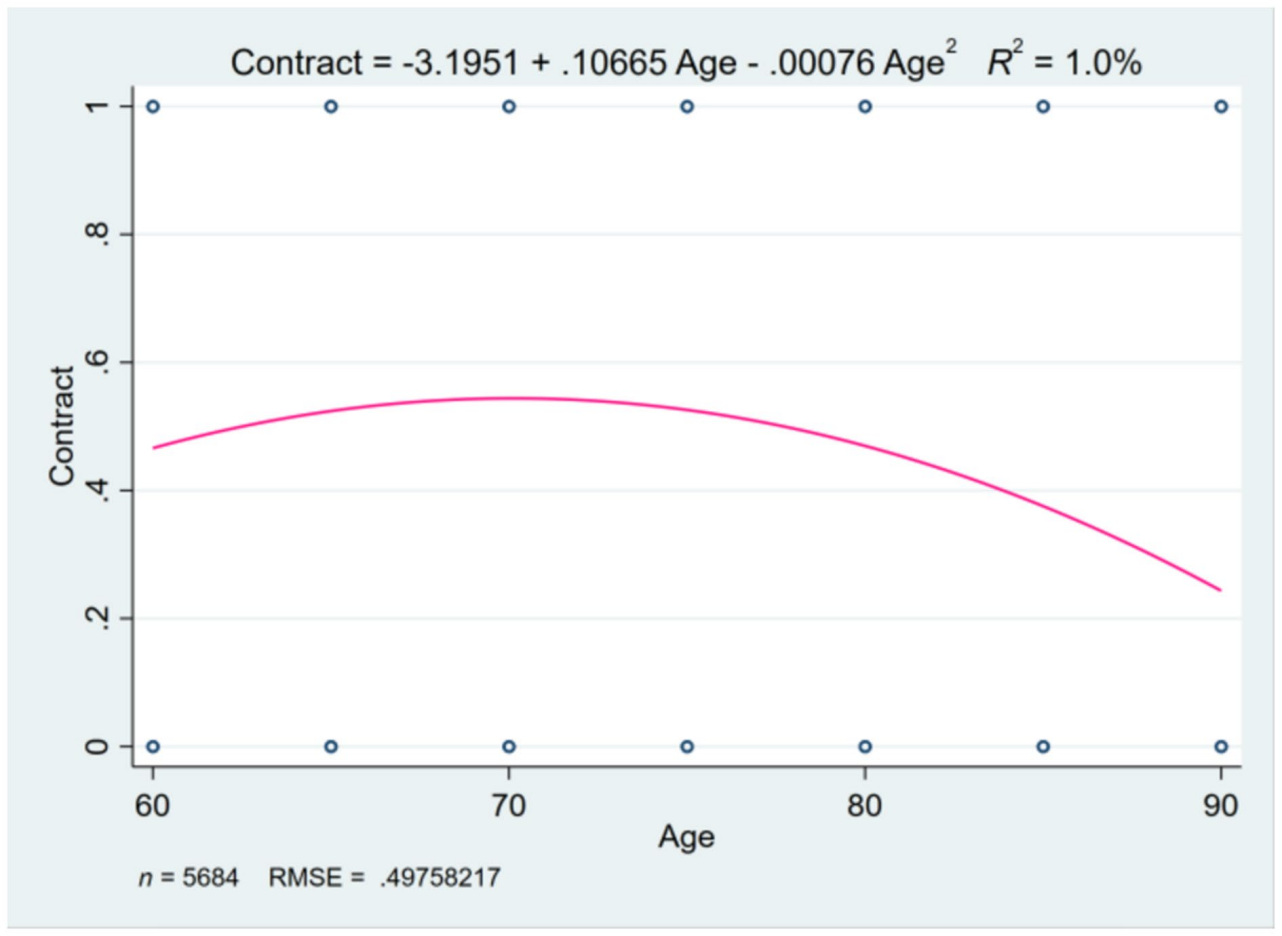
The inverted U-shaped curve of the impact of age on contracting behavior.

By plotting the fitted curve of the relationship between age and contracting behavior, an inverted U-shaped trend was observed. This trend indicated that for individuals aged 60–90, the contracting rate for family doctor services initially rises and then declines. The upward phase of the curve reflected a higher propensity for younger seniors to contract with family doctors. A peak in the middle of the curve suggested that there is a specific age range where the probability of contracting is highest. According to the fitted curve equation, the peak value is 70 years old. After the age of 70, the behavior of signing contracts for family doctor services gradually decreases as illustrated in Figure 2.

## 4 Discussion

As the aging of China’s population deepens, proactively addressing demographic aging has ascended to a national strategic priority, with health issues among older adults emerging as a significant societal concern. With advancing age, there is a progressive decline in the health status of older adults (30, 31). The family doctor contract system represents an effective approach to meet the healthcare needs of older adults, enhance their health status, reduce the economic burden of disease, and improve the efficiency of medical resource utilization (32, 33).

This study, based on statistical data from the health information system of the Wangying Community Health Service Center in Huai’an, employs propensity score matching and multiple logistic regression analysis to explore the factors influencing the signing of family doctor services among older adults. It was found that approximately 50.00% of older adults are signed up with a family doctor, with age, the number of medical visits, and the number of chronic diseases being key factors affecting signing. Another significant finding was that age is significantly correlated with the signing behavior for family doctor services among older adults, displaying an inverted “U” trend with increasing age. This indicated that both younger and older seniors have relatively low rates of signing for family doctor services. This is mainly because younger seniors generally have better overall health status, thus their demand for healthcare services is not urgent. Simultaneously, this group may lack awareness of the importance of long-term health management and preventative care, which leads to a diminished sensitivity and regard for the value and necessity of signing up for family doctor services. As age increases, the health of older adults gradually declines and the incidence of chronic diseases rises, leading to increased demand for medical resources. Therefore, for middle-aged seniors, the attractiveness of family doctor services may increase as their health status changes. This segment of the population may place more emphasis on regular health check-ups, disease prevention, and professional health management services, engaging in healthcare behaviors through medical means, thereby leading to an increase in the behavior of signing with family doctors (34). In contrast, older seniors, due to further deterioration in their health, may face more health challenges, such as the worsening of chronic diseases and an increased risk of disability. This theoretically should heighten their needs for family doctor services (35, 36).

However, their utilization of family doctor services may be hindered by a variety of factors. These include insufficient knowledge of the healthcare system and insurance reimbursement policies, limited understanding of the services’ content and benefits, as well as objective challenges such as financial burdens, complex medical processes, and unfamiliarity with electronic medical applications. These barriers may even intensify with advancing age (37). Meanwhile, neglect of long-term health management and preventive care, as well as societal and cultural taboos surrounding diseases and death, may also prevent them from proactively anticipating and planning for potential health risks, thus failing to sign up for family doctor services (38). However, we must acknowledge that family doctors and community health service centers primarily serve the functions of disease prevention, monitoring, and preliminary diagnosis, and are limited in their capacity to conduct in-depth, specialized diagnostics and treatments (39). As age increases and health conditions and related diseases deteriorate, older residents may be more inclined to seek treatment at specialized and higher-tier hospitals to access more professional services, thereby improving their health status. This may also lead to a decrease in the signing and utilization of family doctor services among older adults.

The number of medical visits is also a crucial factor influencing the contract rates of family doctor services among older adults. This is predominantly because the number of medical visits accurately reflects the healthcare needs of older adults—those with higher numbers of visits generally require more extensive healthcare and related services, hence showing higher rates of contract engagement. Moreover, as the number of visits increases, older adults’ familiarity with relevant service policies tends to improve thanks to ongoing education provided by healthcare professionals (40). Consequently, their trust in and reliance on family doctors also tend to grow (41), further augmenting their demand for and utilization of family doctor services.

Interestingly, this study found that the contracting rates for family doctor services decreases as the number of chronic diseases an older adult has increases. This may be due to the complexity of medical needs brought about by comorbid chronic diseases (42). For example, older adults with acute coronary syndromes (ACSs) often have multiple chronic conditions (MCCs). In addition to traditional cardiovascular risk factors (such as hypertension, hyperlipidemia, and diabetes), common cardiovascular comorbidities include heart failure, stroke, and atrial fibrillation, while prevalent non-cardiovascular comorbidities include chronic kidney disease, anemia, depression, and chronic obstructive pulmonary disease. Their healthcare needs are complex, and the presence of MCCs affects ACS presentation (such as increasing type 2 myocardial infarction occurrences), clinical course, and prognosis, often requiring specialists to create personalized treatment plans (43). In this context, older adults with multiple chronic conditions may require more comprehensive, multidisciplinary specialized treatment, which family doctors cannot fully provide. As a result, older adults with multiple chronic diseases are more inclined to seek help directly from specialists.

Additionally, the management and coordination of care for older adults with multiple chronic diseases require more time and effort. Currently, the family doctor contract system in China primarily operates under a model where one family doctor manages multiple patients (44). This creates challenges as family doctors may lack the time and resources to adequately manage the complex health needs of older adults with multiple chronic conditions. This often leads to a decrease in the willingness of older adults with such conditions to sign contracts with family doctors, as their medical needs exceed the scope of basic care.

To specifically enhance the contracting rates for family doctor services among older adults, we believe interventions can be primarily approached from four aspects.

First of all, to enhance the quality of family doctor contract services, expanding the scope of these services, and refining the utilization processes to better accommodate older adults is imperative. As the cornerstone of primary healthcare, the professional skills and service attitudes of family doctors directly impact the quality of service provided (41). Continuous education and training should be implemented to enhance family doctors’ proficiency in managing common and chronic diseases, health promotion, and disease prevention, ensuring high-quality service delivery. Emphasis on service attitude is crucial, particularly in providing patient and supportive care to older patients. Moreover, optimizing the design of contract service packages is essential. Family doctor service contracts should be tailored to reflect the health conditions and preferences of older adults, creating suitable conditions for their healthcare needs.

There is a need to strengthen service provision across different age groups, offering more comprehensive contract services. For younger, healthier older adults, it is essential to strengthen policy promotion and raise public awareness among this group. Communities can regularly organize policy briefings and training sessions, inviting professionals from healthcare institutions and insurance companies to provide face-to-face explanations and answer questions. Additionally, community senior activity centers and neighborhood committees can serve as platforms to directly promote the family doctor contract system to residents. Various media channels such as television, radio, newspapers, and social media can also be utilized to conduct comprehensive publicity for family doctor policies. By producing engaging promotional videos, special reports, and educational articles, the family doctor system can be introduced to younger older adults and their families, encouraging active participation. Furthermore, it is important to expand related services to meet the needs of this demographic, including providing routine health care and disease prevention services to address their current primary needs. For older adults with poor health conditions, especially older adults over the age of 70, the following measures can be considered when providing family doctor contract services: Initially, develop personalized medical plans to ensure assessment and management based on their specific health needs. Secondly, family doctors should collaborate with multidisciplinary teams to integrate various medical resources for comprehensive care. Additionally, strengthen home healthcare services and in-home nursing to facilitate access to necessary medical support for older adults. Health education and self-management guidance should also be provided to help them raise health awareness and develop disease management skills. Furthermore, simplifying the medical process, reducing medical costs for older adults, and lowering the barriers to accessing medical services are also necessary measures (45, 46). Finally, service packages should be priced reasonably based on the different ages and needs of older adults, optimizing payment costs and insurance reimbursement rates to encourage more residents to utilize family doctor contract services. This ensures coverage of service costs without imposing financial burdens on residents. Collaboration with insurance companies to reasonably increase the reimbursement rates for family doctor services can reduce the out-of-pocket costs for residents, thereby enhancing the accessibility of services.

Secondly, it is crucial to intensify the promotion of family doctor contract services and refine related incentive as well as guarantee policies to enhance the policy’s guidance and targeting. Research has indicated that awareness of family doctor contract services is a key factor affecting the contracting rates, suggesting that the promotional efforts for these services need to be strengthened (17, 47, 48). Targeted promotional efforts should be made for older adults at different age stages and with varying health conditions, informing them how family doctor services can meet their routine medical needs, thereby increasing the contracting rates for these services. Therefore, establishing connections with older adults and their families, building good doctor-resident relationships, and fostering positive interactions are essential. This would enhance older adults’ sense of social trust and participation, guiding them to engage in family doctor services (49, 50). Additionally, in the digital era, older adults have access to a more diverse range of information sources. It is essential to fully leverage the convenience and broad reach of digital communication to enhance the promotional channels for family doctor contract services. By effectively utilizing personal networks and internet platforms, promotional efforts can be expanded to older adults and their families, thereby increasing awareness of family doctor contract services (51).

Thirdly, enhancing the promotion of medical and health knowledge, improving citizens’ health literacy and awareness of health risks, and actively responding to population aging are crucial. Health education is an important component of health management, yet its importance is still not sufficiently recognized across different levels of healthcare institutions. There is a contradiction between the weak capacity for health education in primary healthcare sectors and the gap in needs for public health education (52). Appropriate channels for health knowledge dissemination should be established to improve residents’ health literacy and strengthen their focus on long-term health. Health education should be demand-driven for older adults at different stages of life, with an emphasis on digital health education and promotion. This approach aims to enhance the health literacy and health risk prevention awareness of older adults and their families, encouraging them to actively use primary healthcare resources, thereby increasing the contracting rates for family doctor services (53). Health education initiatives, participation in activities, and other forms should be integrated into the daily lives of older adults to more comprehensively and promptly respond to their everyday medical and health needs. This will enhance the accessibility of family doctor services for older adults, thus improving the health status of especially older seniors, increasing older adults’ life satisfaction, improving the utilization rate of social medical resources, reducing unreasonable medical expenditures, and alleviating the medical burden on individuals, families and society.

Fourthly, refining supportive policies and advancing the development of a healthy aging society is essential. Improve financial subsidies, tax incentives, and career development paths to attract more skilled doctors to primary healthcare services. At the same time, fully utilize the role of family doctor contract services in the home-based community older adult care model and, in conjunction with long-term care insurance, integrate and mobilize grassroots older adult care and medical service resources are essential measures. Moreover, establishing good interactions with older adults and their communities are also crucial steps in building a healthy aging society (54). All these suggestions aim to address key gaps in family doctor service utilization and support more effective policy interventions.

## 5 Conclusion

The intensification of population aging underscores the urgency of the healthcare service crisis. Family doctor contract services, as a core component of the primary healthcare system, play an irreplaceable role in improving the health of older adults. This study investigated the contracting of family doctor services by older adults at the Wangying Community Health Service Center in Huai’an, Jiangsu Province, China, and its influencing factors. The findings revealed that age, the number of medical visits, and the number of chronic diseases significantly impact the willingness of older adults to sign contracts. Notably, there is an inverted U-shaped relationship between age and the willingness to contract. This study employed primary survey microdata for analysis, allowing for a more detailed and accurate examination of the factors influencing the contract rate of family doctor services and reflecting the unique challenges and needs older adults may face in utilizing these services. vGiven these findings, policymakers should urgently consider reforms to the family doctor contracting policy to address the gaps revealed by this research. Specifically, adjustments should be made to enhance accessibility for older adults with multiple chronic conditions, who may be less inclined to participate in such services. Additionally, efforts to encourage frequent users of healthcare services to contract with family doctors should be strengthened, as higher visit frequencies are positively correlated with contracting behavior. Policymakers must also address the declining willingness to contract beyond a certain age, ensuring that the oldest and potentially most vulnerable individuals are not left without adequate primary care support. By refining family doctor policies in these areas, healthcare systems can better respond to the aging population and improve outcomes for older adults.

However, this study had some limitations, Firstly, the contracting behavior of older adults for family doctor services is influenced by a multitude of factors, including but not limited to socioeconomic status, cultural background, health awareness, living environment, accessibility of medical resources, and policy support. These factors collectively affect older adults’ perceptions, attitudes, and behaviors toward family doctor services, thus influencing their contracting behavior. Future research should delve deeper into these multidimensional factors to uncover the mechanisms by which various factors influence older adults’ contracting behaviors. This comprehensive perspective will advance the optimization of family doctor service policies and promote the development of healthy aging. Secondly, the study’s another limitation stems from its reliance on cross-sectional data, which captures a snapshot of circumstances at a specific time. Consequently, it does not allow for an analysis of the dynamic impacts of factors such as gender, age, the number of medical visits, and the number of chronic diseases on older adults’ willingness to engage in family doctor services. Future research should investigate whether the effects of these factors are influenced by time-related changes.

## Data Availability

The raw data supporting the conclusions of this article will be made available by the authors, without undue reservation.
